# Learning Predictive Statistics: Strategies and Brain Mechanisms

**DOI:** 10.1523/JNEUROSCI.0144-17.2017

**Published:** 2017-08-30

**Authors:** Rui Wang, Yuan Shen, Peter Tino, Andrew E. Welchman, Zoe Kourtzi

**Affiliations:** ^1^Key Laboratory of Mental Health, Institute of Psychology, Chinese Academy of Sciences, Beijing 100101, China,; ^2^Department of Psychology, University of Cambridge, Cambridge CB2 3EB, United Kingdom,; ^3^Department of Mathematical Sciences, Xi'an Jiaotong-Liverpool University, Suzhou 215123, China, and; ^4^School of Computer Science, University of Birmingham, Birmingham B15 2TT, United Kingdom

**Keywords:** fMRI, learning, prediction, vision

## Abstract

When immersed in a new environment, we are challenged to decipher initially incomprehensible streams of sensory information. However, quite rapidly, the brain finds structure and meaning in these incoming signals, helping us to predict and prepare ourselves for future actions. This skill relies on extracting the statistics of event streams in the environment that contain regularities of variable complexity from simple repetitive patterns to complex probabilistic combinations. Here, we test the brain mechanisms that mediate our ability to adapt to the environment's statistics and predict upcoming events. By combining behavioral training and multisession fMRI in human participants (male and female), we track the corticostriatal mechanisms that mediate learning of temporal sequences as they change in structure complexity. We show that learning of predictive structures relates to individual decision strategy; that is, selecting the most probable outcome in a given context (maximizing) versus matching the exact sequence statistics. These strategies engage distinct human brain regions: maximizing engages dorsolateral prefrontal, cingulate, sensory–motor regions, and basal ganglia (dorsal caudate, putamen), whereas matching engages occipitotemporal regions (including the hippocampus) and basal ganglia (ventral caudate). Our findings provide evidence for distinct corticostriatal mechanisms that facilitate our ability to extract behaviorally relevant statistics to make predictions.

**SIGNIFICANCE STATEMENT** Making predictions about future events relies on interpreting streams of information that may initially appear incomprehensible. Past work has studied how humans identify repetitive patterns and associative pairings. However, the natural environment contains regularities that vary in complexity from simple repetition to complex probabilistic combinations. Here, we combine behavior and multisession fMRI to track the brain mechanisms that mediate our ability to adapt to changes in the environment's statistics. We provide evidence for an alternate route for learning complex temporal statistics: extracting the most probable outcome in a given context is implemented by interactions between executive and motor corticostriatal mechanisms compared with visual corticostriatal circuits (including hippocampal cortex) that support learning of the exact temporal statistics.

## Introduction

Making predictions about future events challenges us to extract structure from streams of sensory signals that initially appear incomprehensible. Typically, event structures in the natural environment contain regularities of variable complexity from simple repetitive patterns to more complex probabilistic combinations. For example, when learning a new piece of music or a new language, we extract simple repetitive patterns (e.g., tones, syllables) and more complex contingencies (e.g., melodies or phoneme pairs) that determine the probability with which events occur. Learning to extract these statistics allows us to interpret incoming signals rapidly and predict upcoming events. Despite the fundamental importance of this type of statistical learning for sensory interpretation and prediction, we know surprisingly little about its neural basis.

Previous work on statistical learning has focused on simple repetitive patterns or associative pairings. Behavioral studies provide evidence that mere exposure (i.e., without explicit feedback) to co-occurring stimuli can drive learning of contingencies (for reviews, see Perruchet and Pacton, 2006; Aslin and Newport, 2012). For example, observers become familiar with structured patterns after exposure to items (e.g., shapes, tones, or syllables) that co-occur spatially or appear in a temporal sequence (Saffran et al., 1999; Chun, 2000; Fiser and Aslin, 2002; Turk-Browne et al., 2005). Here, we investigate the functional brain mechanisms that mediate our ability to adapt to changes in the environment's statistics and learn behaviorally relevant structures for making predictions.

**Figure 1.**
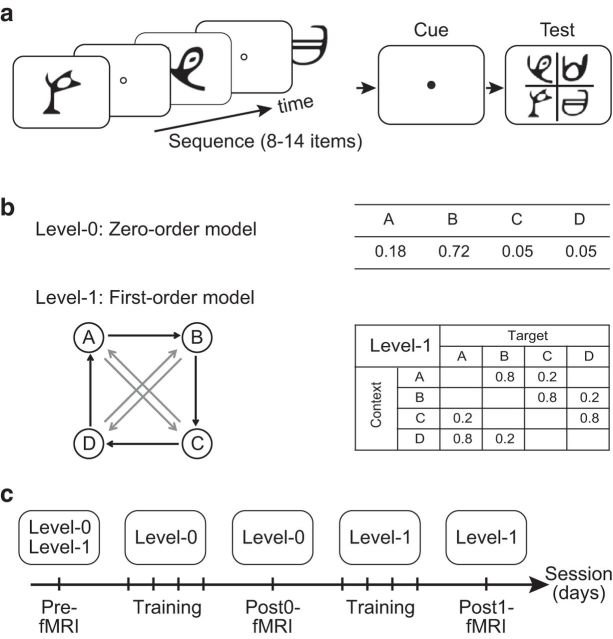
Trial and sequence design. ***a***, Trial design. 8–14 stimuli were presented sequentially, followed by a cue and the test display. ***b***, Sequence design. Markov models comprised two levels of complexity. For the zero-order model (Level 0), different states (***A***–***D***) are assigned to four symbols with different frequencies. For the first-order model (Level 1), a diagram indicates states (circles) and transitional probabilities (black arrow: high probability, e.g., 80%; gray arrow: low probability, e.g., 20%). Transitional probabilities are shown in a four-by-four conditional probability matrix, with rows indicating temporal context and columns indicating the corresponding target. ***c***, Experimental protocol. Observers underwent multiple days of behavioral training first with zero-order sequences and then with first-order sequences. For each level, observers completed three to five training sessions (an average of four sessions is shown for illustration purposes). Three fMRI scanning sessions were conducted before (Pre) and immediately after training per level (Post0, Post1).

We combine behavioral measures with multisession fMRI (before and after training) to examine the neural mechanisms that mediate learning of temporal sequences that change in their statistics from repetitive patterns to more complex probabilistic contingencies. To do so unencumbered by past experience, we tested participants with sequences of unfamiliar symbols, in which the complexity of the sequence structure changed unbeknownst to the participants (see Fig. 1). We increased sequence complexity by manipulating the memory order (i.e., context length) of the Markov model used to generate the sequences. In particular, we presented participants first with sequences that were determined by frequency statistics (i.e., occurrence probability per symbol) and then by more complex, context-based statistics (i.e., the probability of a given symbol appearing depends on the preceding symbol). Participants performed a prediction task in which they indicated which symbol they expected to appear after exposure to a sequence of variable length. Following previous statistical learning paradigms, participants were exposed to the sequences without trial-by-trial feedback.

Our behavioral results show that individuals adapt to the environment's statistics; that is, they are able to extract predictive structures of different complexity. Further, we show that learning of predictive structures relates to individual decision strategy; that is, individuals differed in their decision strategies, favoring either probability maximization (i.e., extracting the most probable outcome in a given context) or matching the exact sequence statistics. We used this variability in decision strategy to investigate fMRI activity. We find that distinct corticostriatal mechanisms mediate the two strategies: matching engages occipitotemporal regions (including the hippocampus) and ventral caudate, whereas maximizing engages dorsolateral prefrontal, cingulate, sensory–motor regions, and basal ganglia (dorsal caudate, putamen). This provides evidence for differentiated corticostriatal mechanisms that support learning of behaviorally relevant statistics for making predictions.

## Materials and Methods

### Observers

Thirty-four participants (mean age = 21.8 years, male and female) participated in the experiments (main experiment: *n* = 23; control experiment: *n* = 11). The data from two participants were excluded from further imaging analysis due to excessive head movement (>3 mm). All observers were naive to the aim of the study, had normal or corrected-to-normal vision, and gave written informed consent. This study was approved by the University of Birmingham Ethics Committee.

### Stimuli

Stimuli comprised four symbols chosen from the Ndjuká syllabary (Turk-Browne et al., 2009; see Fig. 1*a*). These symbols were highly discriminable from each other and were unfamiliar to the observers. Each symbol subtended 8.5° of visual angle and was presented in black on a midgray background. Experiments were controlled using MATLAB and the Psychophysics toolbox 3 (Brainard, 1997; Pelli, 1997). For the behavioral training sessions, stimuli were presented on a 21-inch CRT monitor (ViewSonic P225f 1280 ×1024 pixel, 85 Hz frame rate) at a distance of 45 cm. For the pretraining and posttraining fMRI scans, stimuli were presented using a projector and a mirror setup (1280 × 1024 pixel, 60 Hz frame rate) at a viewing distance of 67.5 cm. The physical size of the stimuli was adjusted so that angular size was constant during behavioral and scanning sessions.

### Sequence design

To generate probabilistic sequences of different complexity, we used a temporal Markov model and manipulated the memory order of the sequence, which we refer to as the context length.

The Markov model consists of a series of symbols in which the symbol at time *i* is determined probabilistically by the previous *k* symbols. We refer to the symbol presented at time *i*, *s*(*i*), as the target and to the preceding *k*-tuple of symbols (*s*(*i* − 1), *s*(*i* − 2), … *s*(*i* − *k*)) as the context. The value of *k* is the order or level of the following model:

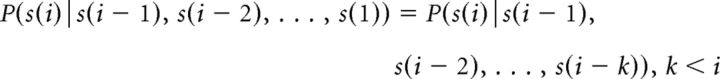
 The simplest *k* = *0*^th^ order model is a random memory-less source. This generates, at each time point *i*, a symbol according to symbol probability *P*(*s*) without taking account of the previously generated symbols.

The order *k* = *1* Markov model generates symbol *s*(*i*) at each time *i* conditional on the previously generated symbol *s*(*i* − 1). This introduces a memory in the sequence; that is, the probability of a particular symbol at time *i* strongly depends on the preceding symbol *s*(*i* − 1). Unconditional symbol probabilities *P*(*s*(*i*)) for the case *k* = *0* are replaced with conditional ones, *P*(*s*(*i*) | *s*(*i* − 1)).

At each time point, the symbol that follows a given context is determined probabilistically, making the Markov sequences stochastic. The underlying Markov model can be represented through the associated context-conditional target probabilities. We used four symbols that we refer to as stimuli A, B, C, and D. The correspondence between stimuli and symbols was counterbalanced across participants.

For Level 0, the Markov model was based on the probability of symbol occurrence: one symbol had a high probability of occurrence, one low probability, whereas the remaining two symbols appeared rarely (see Fig. 1*b*). For example, the probabilities of occurrence for the four symbols A–D were 0.18, 0.72, 0.05, and 0.05, respectively. Presentation of a given symbol was independent of the stimuli that preceded it.

For Level 1, the target depended on the immediately preceding stimulus (see Fig. 1*b*). Given a context (the last seen symbol) only one of two targets could follow; one had a high probability of being presented and the other a low probability (e.g., 80% vs 20%). For example, when symbol A was presented, only symbols B or C were allowed to follow, and symbol B had a higher probability of occurrence than symbol C.

### Task design

We tested learning of temporal structures that differed in their complexity; that is, sequences determined by simple frequency statistics (Level 0) and more complex sequences defined by context-based statistics (Level 1). To define the complexity of our sequences, we quantified the average past–future mutual information in the sequences generated by stochastic sources (Grassberger, 1986), providing a statistic that has been applied in a number of probabilistic contexts (Shaw, 1984; Li, 1991). For Markov models of order 0 or 1, complexity is expressed as the difference between the entropy of the marginal symbol distribution and the entropy rate of the Markov chain (Li, 1991). This measure quantifies the average reduction in uncertainty of the next symbol in a sequence when the memory of the generating source is taken into account. For 0-order Markov models, the complexity is 0 because the source itself is memory-less. For Markov models of order 1, conditioning on the last symbol will reduce the uncertainty. For example, for the first-order Markov model that we used, the marginal symbol probabilities are equal, resulting in entropy close to the maximum value of two bits. However, conditional on the last symbol, only two symbols are allowed with unequal probabilities, resulting in lower entropy rate and therefore higher complexity (1.28).

To investigate whether participants adapt to changes in the temporal structure, we ensured that the sequences across levels were matched for properties (i.e., number or identity of symbols) other than complexity. Further, we designed the stochastic sources from which the sequences were generated so that the context-conditional uncertainty remained highly similar across levels. In particular, for the zero-order source, only two symbols were likely to occur most of the time; the remaining two symbols had very low probability (0.05). This was introduced to ensure that there was no difference in the number of symbols presented across levels. Of the two dominant symbols, one was more probable (probability 0.72) than the other (probability 0.18). This structure is preserved in Markov chain of order 1, in which, conditional on the previous symbols, only two symbols were allowed to follow, one with higher probability (0.80) than the other (0.20). This ensures that the structure of the generated sequences across levels differed predominantly in memory order (i.e., context length) rather than context-conditional probability.

### Procedure

Observers were initially familiarized with the task through a brief practice session (8 min) with random sequences (i.e., all four symbols were presented with equal probability 25% in a random order). After this, observers participated in multiple behavioral training and fMRI scanning sessions that were conducted on different days (see Fig. 1*c*). Participants were trained with structured sequences and tested with both structured and random sequences to ensure that training was specific to the trained sequences.

In the first scanning session, participants were presented with zero- and first-order sequences and random sequences. Observers were then trained with zero-order sequences and subsequently with first-order sequences. For each level, observers completed a minimum of three and a maximum of five training sessions (840–1400 trials). Training at each level ended when participants reached plateau performance (i.e., performance did not change significantly for two sessions). A posttraining scanning session followed training per level (i.e., on the following day after completion of training), during which observers were presented with structured sequences determined by the statistics of the trained level and random sequences. The mean time interval (±SE) between the pretraining session and the final test session was 23.5 ± 0.5 d.

### Psychophysical training

Each training session comprised five blocks of structured sequences (56 trials per block) and lasted 1 h. To ensure that sequences in each block were representative of the Markov model order per level, we generated 10,000 Markov sequences per level comprising 672 stimuli per sequence. We then estimated the Kullback–Leibler (KL) divergence between each example sequence and the generating source. In particular, for Level 0 sequences, this was defined as follows:

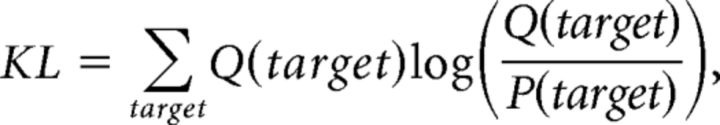
 and for Level 1 sequences, this was defined as follows:

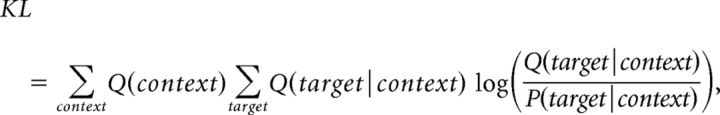
 where *P*( ) refers to probabilities or conditional probabilities derived from the presented sequences and *Q*( ) refers to those specified by the source. We selected 50 sequences with the lowest KL divergence (i.e., these sequences matched closely the Markov model per level). The sequences presented to the participants during the experiments were selected randomly from this sequence set.

For each trial, a sequence of 8–14 stimuli appeared in the center of the screen one at a time in a continuous stream, each for 300 ms followed by a central white fixation dot (interstimulus interval, ISI) for 500 ms (see Fig. 1*a*). This variable trial length ensured that observers maintained attention during the whole trial. Each block comprised equal number of trials with the same number of stimuli. The end of each trial was indicated by a red dot cue that was presented for 500 ms. After this, all 4 symbols were shown in a 2 × 2 grid. The positions of test stimuli were randomized from trial to trial. Observers were asked to indicate which symbol they expected to appear after the preceding sequence by pressing a key corresponding to the location of the predicted symbol. Observers learned a stimulus-key mapping during the familiarization phase: keys 8, 9, 5, and 6 in the number pad corresponded to the four positions of the test stimuli: upper left, upper right, lower left, and lower right, respectively. After the observer's response, a white circle appeared on the selected stimulus for 300 ms to indicate the observer's choice, followed by a fixation dot for 150 ms (intertrial interval) before the start of the next trial. If no response was made within 2 s, a null response was recorded and the next trial started. Participants were given feedback (i.e., score in the form of PI, see “Data analysis” section) at the end of each block, rather than per-trial error feedback, which motivated them to continue with training.

### Scanning sessions

The pretraining scanning session (Pre) included six runs (i.e., three runs per level), the order of which was randomized across participants. Scanning sessions after training per level (denoted as Post0, Post1) included nine runs of structured sequences determined by the same statistics as the corresponding trained level and random sequences. Each run comprised five blocks of structured and five blocks of random sequences presented in a random counterbalanced order (two trials per block; a total of 10 structured and 10 random trials per run), with an additional two 16 s fixation blocks, one at the beginning and one at the end of each run. Each trial comprised a sequence of 10 stimuli that were presented for 250 ms each, separated by a blank interval during which a white fixation dot was presented for 250 ms. After the sequence, a fixation screen (central red dot) appeared for 4 s before the test display (comprising 4 test stimuli) appeared for 1.5 s. Observers were asked to indicate which symbol they expected to appear after the preceding sequence by pressing a key corresponding to the location of the predicted symbol. A white fixation was then presented for 5.5 s before the start of the next trial. In contrast to the training sessions, no feedback was given during scanning.

### fMRI data acquisition

The experiments were conducted at the Birmingham University Imaging Centre using a 3 T Philips Achieva MRI scanner. T2*-weighted functional and T1-weighted anatomical (1 × 1 × 1 mm resolution, slices = 175) data were collected with a 32-channel SENSE head coil. Echoplanar imaging data (gradient echo pulse sequences) were acquired from 32 slices (whole-brain coverage; TR = 2000 ms; TE = 35 ms; 2.5 × 2.5 × 4 mm resolution).

### Behavioral data analysis

#### 

##### Performance index (PI).

We assessed participant responses in a probabilistic manner. For each context, we computed the absolute Euclidean distance between the distribution of participant responses and the distribution of presented targets estimated across 56 trials per block as follows:

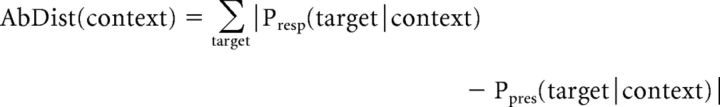
 where the sum is over targets from the symbol set A–D. We estimate AbDist per context for each block. We quantified the minimum overlap between these two distributions by computing a Performance Index (PI) per context as follows:

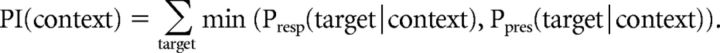
 Note that PI(context) = 1 − AbDist(context)/2. The overall PI is then computed as the average of the performance indices across contexts, PI(context), weighted by the corresponding stationary context probabilities as follows:

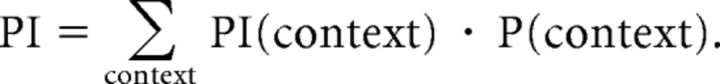
 To compare across different levels, we defined a normalized PI measure that quantifies participant performance relative to random guessing. We computed a random guess baseline, PI_rand_, which reflects participant responses to targets with equal probability of 25% for each target per trial for Level 0, (PI_rand_ = 0.53) and equal probability for each target for a given context for Level 1 (PI_rand_ = 0.45). To correct for differences in random guess baselines across levels, we subtracted the random guess baseline from the PI (PI_normalized_ = PI − PI_rand_).

##### Strategy choice and strategy index.

To quantify each observer's strategy, we compared individual participant response distributions (response-based model) to two baseline models: (1) probability matching, in which probability distributions are derived from the Markov models that generated the presented sequences (model matching), and (2) a probability maximization model, in which only the single most likely outcome is allowed for each context (model maximization). We used KL divergence to compare the response distribution to each of these two models. KL is defined as follows:

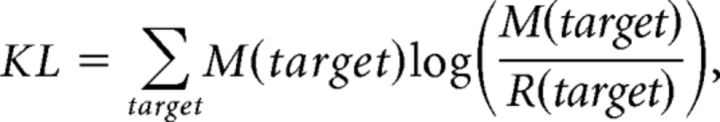
 for the Level 0 model and

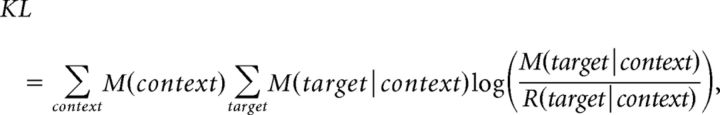
 for the Level 1 model, where *R*( ) and *M*( ) denote the probability distribution or conditional probability distribution derived from the human responses and the models (i.e., probability matching or maximization), respectively, across all the conditions.

We quantified the difference between the KL divergence from model matching to the response-based model and the KL divergence from model maximization to the response-based model. We refer to this quantity as strategy choice indicated by ΔKL (model maximization, model matching). Negative strategy choice values indicate a strategy closer to matching, whereas positive values indicate a strategy closer to maximization. We computed strategy choice per training block, resulting in a strategy curve across training for each individual participant. We then derived an individual strategy index by calculating the integral of each participant's strategy curve and subtracting it from the integral of the exact matching curve, as defined by model matching across training. We defined the integral curve difference between individual strategy and exact matching as the individual strategy index. We used this index to investigate the relationship of individual strategy and fMRI signals.

### fMRI data analysis

#### 

##### Data preprocessing.

MRI data were processed using Brain Voyager QX (Brain Innovation). T1-weighted anatomical data were used for coregistration, 3D cortex reconstruction, inflation, and flattening. Preprocessing of the functional data involved slice scan time correction, head motion correction, temporal high-pass filtering (three cycles), and removal of linear trends. Spatial smoothing (Gaussian filter; 5 mm FWHM kernel) was performed for group random-effect analysis. The functional images were aligned to anatomical data and the complete data were transformed into Talairach space. For each observer, the functional imaging data between sessions were coaligned by registering all volumes of each observer to the first functional volume acquired during the first session.

##### Whole-brain general linear model (GLM).

BOLD responses for each trial comprising structured or random sequences were modeled separately for each session using a GLM. To search for brain regions that showed learning-dependent changes across sessions, we constructed a multiple regression design matrix that included the two stimulus conditions (structured vs random sequences) for each of the scanning sessions (Pre, Post0, Post1) as regressors. Each regressor was time locked to trial onset and included a range of volumes (see Figs. 3 and 4: five volumes and Fig. 5*b*: three volumes). To remove residual motion artifacts, the six zero-centered head movement parameters were also included as regressors. Serial correlations were corrected using a second-order autoregressive model AR(2). The resulting parameter estimates (β value) were used in a voxelwise mixed-design ANOVA with sequence (structured vs random) and scanning session (Pre, Post0, Post1). Statistical maps were cluster threshold corrected (*p* < 0.005) using Monte Carlo simulations (5000 iterations; Forman et al., 1995; Goebel et al., 2006) for multiple-comparison correction that confirmed a familywise error threshold of *p* = 0.05. Note that our results also hold for a more conservative threshold (*p* < 0.001), as recommended by recent studies (Woo et al., 2014; Eklund et al., 2016), but small-volume correction is required for small structures (i.e., putamen) at this threshold.

##### Covariance analysis.

To examine the relationship between brain activation and observers' performance, we conducted a voxelwise covariance analysis. In particular, we used individual strategy index as covariate in a GLM model of fMRI responses. That is, for each voxel, we correlated fMRI signal difference between structured and random sequences before versus after training with the strategy index. We calculated a Pearson correlation coefficient (*R*) for each voxel across the whole brain and identified voxel clusters showing significant correlations (*p* < 0.05, cluster threshold corrected). Positive correlations indicate increased activations after training that relate to maximization, whereas negative correlations indicate increased activations after training that relate to matching because a negative strategy index indicates matching.

## Results

### Behavioral results

Previous studies have compared learning of different spatiotemporal contingencies in separate experiments across different participant groups (Fiser and Aslin, 2002, 2005). Here, to investigate whether individuals extract changes in structure, we presented the same participants with sequences that changed in complexity unbeknownst to them (Fig. 1*a*). We parameterized sequence complexity based on the memory order of the Markov models used to generate the sequences (see Materials and Methods); that is, the degree to which the presentation of a symbol depended on the history of previously presented symbols (Fig. 1*b*). We first presented participants with simple zero-order sequences (Level 0), followed by more complex first-order sequences (Level 1; Fig. 1*c*) because previous work has shown that temporal dependencies are more difficult to learn as their length increases (van den Bos and Poletiek, 2008) and training with simple dependencies may facilitate learning of more complex contingencies (Antoniou et al., 2016). Zero-order sequences (Level 0) were context-less; that is, the presentation of each symbol depended only on the probability of occurrence of each symbol. For first-order sequences (Level 1), the presentation of a particular symbol was conditionally dependent on the previously presented symbol (i.e., context length of one).

Because the sequences we used were probabilistic, we developed a probabilistic measure to assess participants' performance in the prediction task. Specifically, we computed a Performance Index (PI) that indicates how closely the distribution of participant responses matched the probability distribution of the presented symbols. This is preferable to a simple measure of accuracy because the probabilistic nature of the sequences means that the “correct” upcoming symbol is not uniquely specified; therefore, designating a particular choice as correct or incorrect is often arbitrary.

**Figure 2.**
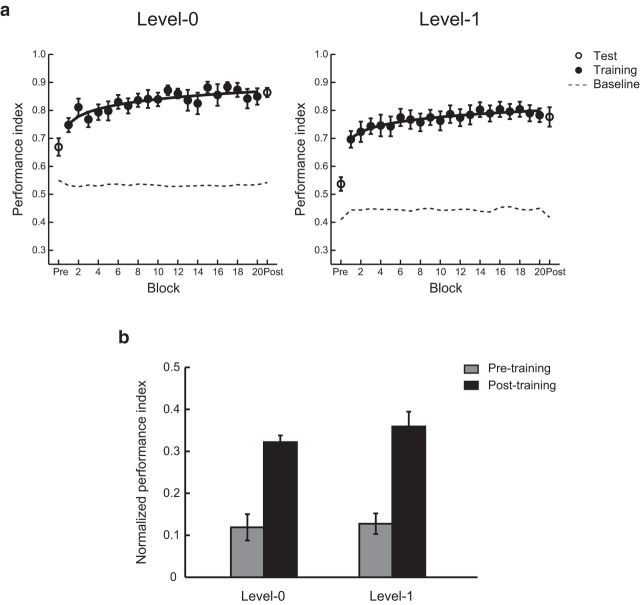
Behavioral performance. ***a***, Mean PI across participants for test (open symbols) and training (solid symbols) blocks for Level 0 and Level 1. Data are fitted (least-squares nonlinear fit) across training blocks. Random guess baseline is indicated by dotted lines. ***b***, Normalized PI during scanning. Data are shown before (gray bars) and after (black bars) training for each level. Error bars indicate SEM.

Comparing normalized performance (i.e., after subtracting performance based on random guessing) before and after training per level (Fig. 2) showed that observers improved substantially and learned the probabilistic structures (i.e., mean improvement >20% for both levels). A repeated-measures ANOVA with session (Pre, Post) and level (Level 0, Level 1) showed a significant effect of session (*F*_(1,20)_ = 82.0, *p* < 0.001), but no significant effect of level (*F*_(1,20)_ < 1, *p* = 0.358) and no significant interaction (*F*_(1,20)_ < 1 *p* = 0.664), indicating that observers improved similarly at both levels through training. Interestingly, performance during the pretraining test session was higher than random guessing (*F*_(1,20)_ = 42.8, *p* < 0.001), suggesting that fast learning of structured sequences is consistent with the learning time course reported in previous perceptual learning studies (Karni and Sagi, 1993). However, improvement continued during training across blocks; that is, mean performance for the last two training blocks was significantly higher than the mean performance for the first two training blocks (*F*_(1,20)_ = 12.8, *p* = 0.002).

We then tested whether this learning-dependent improvement was specific to the trained structured sequences. First, we compared performance accuracy (i.e., proportion of correctly predicted trials based on the predefined sequences) for structured and random sequences. A repeated-measures ANOVA showed a significant interaction of Session (Pre, Post) and sequence type (structured vs random) for Level 0 (*F*_(1,20)_ = 24.1, *p* < 0.001) and Level 1 (*F*_(1,20)_ = 54.5, *p* < 0.001), suggesting that learning improvement was specific to structured sequences. Second, we conducted a no-training control experiment, during which participants (*n* = 11) were tested in two separate behavioral sessions but did not participate in any training sessions. The two test sessions were spaced apart by a period (27.9 ± 1.9 days on average), comparable to the main experiment (23.5 days on average). Our results showed that there were no significant differences in performance between the two test sessions. In particular, a repeated-measures ANOVA with session (Session 1, Session 2) and Level (Level 0, Level 1) did not show any significant effect of session (*F*_(1,10)_ < 1, *p* = 0.736) or level (*F*_(1,10)_ = 1.84, *p* = 0.205) and no significant interaction (*F*_(1,10)_ = 1.16, *p* = 0.308). These results suggest that the improvement that we observed in the main experiment was specific to training, rather than simply being due to repeating the test session twice (before and after training). Comparing PI between experiments (main vs no-training control experiment) showed a significant interaction between experiment and session (Level 0: *F*_(1,30)_ = 15.1, *p* = 0.001, Level 1: *F*_(1,30)_ = 7.95, *p* = 0.008), consistent with training-induced behavioral improvement.

### fMRI analysis: learning-dependent activation changes

**Figure 3.**
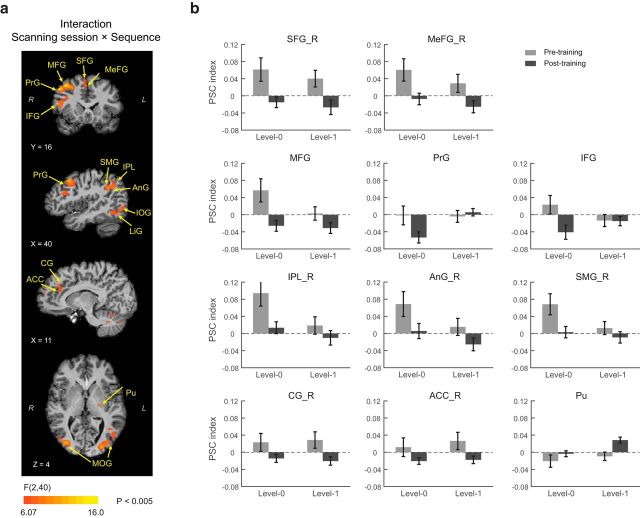
fMRI results. ***a***, GLM maps for the two-way interaction between scanning session (Pre, Post0, Post1) and sequence (structured vs random), at *p* < 0.005 (cluster threshold corrected). Only the first five volumes were included in the analysis that correspond to the presentation of sequence, the participants' prediction, and the test display presentation to avoid confounding the results by the participants' response. Similar results were observed at a more conservative threshold (*p* < 0.001), but small volume correction was necessary for small structures (i.e., putamen) at this threshold. ***b***, PSC index (percentage signal change for structured sequences compared with random sequences) before and after training for Level 0 and Level 1. Data are shown for ROIs that showed a significant interaction between session (pretraining vs posttraining) and sequence (structured vs random). Error bars indicate SEM. Note that different number of runs were scanned before and after training (i.e., pretraining scan comprised three runs per level, whereas posttraining scans comprised nine runs per level). To compare equal amounts of data before and after training, we selected three of the nine runs from each posttraining scan; that is, we divided each session into two time periods and selected randomly one run per time period to match the order in which data were collected during the pretraining scan. Whole-brain voxelwise GLM analysis showed significant interactions for sequence (structured vs random) and scanning session (Pre, Post0, Post1) in the frontal, parietal, and subcortical regions, which is consistent with our main result.

To investigate the brain mechanisms that mediate our ability to adapt to changes in temporal statistics, we performed fMRI on participants before and after training on each level with structured and random sequences. To assess learning-dependent changes in fMRI signals, we conducted a whole-brain voxelwise GLM analysis (RFX group analysis). In particular, we tested for brain regions that showed a significant interaction (*p* < 0.005, cluster threshold corrected) between sequence (structured vs random) and scanning session (Pre, Post0, Post1). This analysis revealed a network of dorsal frontal, cingulate, posterior parietal, occipital, and temporal regions, as well as subcortical (basal ganglia) and cerebellar regions (Fig. 3*a*, Table 1).

**Table 1.** Brain regions showing significant interaction between scanning session (Pre, Post0, Post1) and sequence (structured vs random), *p* < 0.005, cluster corrected

ROI	Hemisphere	Volume (mm^3^)	Peak *X*	Peak *Y*	Peak *Z*	*F*	*p*
Frontal							
SFG	R	1633	36	16	46	15.49539	0.00001
MeFG	R	922	6	32	37	9.33239	0.00047
MFG	L	251	−45	0	37	13.73743	0.00003
MFG	R	4352	45	14	40	17.07472	0.00000
IFG	L	273	−45	2	31	11.74197	0.00010
IFG	R	510	48	14	19	10.29143	0.00025
PrG	L	1462	−45	−4	40	17.85552	0.00000
PrG	R	272	43	15	40	12.12258	0.00008
Insula	L	182	−39	−4	−2	13.93199	0.00003
Insula	R	81	44	14	17	7.47606	0.00174
Parietal							
PCu	L	1381	−21	−64	40	9.97693	0.00031
SPL	L	506	−24	−58	40	11.28717	0.00013
IPL	R	859	39	−50	34	11.30387	0.00013
AnG	R	365	39	−58	34	10.92339	0.00016
SMG	R	148	39	−49	34	11.47595	0.00012
Occipital							
MOG	L	2574	−27	−82	−5	19.95821	0.00000
MOG	R	1263	35	−80	1	12.63784	0.00006
IOG	L	929	−36	−73	−8	21.95450	0.00000
IOG	R	497	37	−79	1	13.67147	0.00003
LiG	L	1346	−35	−70	−6	17.45473	0.00000
LiG	R	759	30	−76	1	11.94279	0.00009
Cuneus	L	293	−24	−82	10	9.99664	0.00030
Cuneus	R	154	24	−79	16	8.28265	0.00098
FG	L	1901	−36	−73	−9	21.95450	0.00000
FG	R	650	36	−63	−5	12.01979	0.00008
Temporal							
MTG	L	662	−41	−58	−4	17.05987	0.00000
ITG	L	516	−44	−58	−5	15.70175	0.00001
SGL	L	81	−42	−51	−3	8.82149	0.00067
PHG	L	149	−39	−50	1	9.78701	0.00035
PHG	R	98	33	−55	−5	11.96719	0.00008
Limbic							
CG	R	188	24	11	43	9.27872	0.00049
ACC	R	160	15	32	22	8.12427	0.00109
Subcortical							
Claustrum	L	132	−37	−4	−2	11.50993	0.00011
Putamen	L	93	−24	−16	4	8.27780	0.00098
Thalamus	L	266	−12	−19	7	8.50720	0.00084
Cerebellum							
Culmen	L	61	−1	−61	−22	7.37876	0.00187
Culmen	R	611	19	−58	−19	11.72096	0.00010
Nodule	L	505	0	−53	−26	16.10464	0.00001
Nodule	R	582	0	−52	−26	16.60786	0.00001
Pyramis	L	197	0	−67	−26	12.81382	0.00005
Pyramis	R	252	6	−70	−26	14.53668	0.00002
Declive	L	586	−36	−61	−11	15.01214	0.00001
Declive	R	1752	18	−58	−17	12.49729	0.00006
Uvula	L	266	0	−68	−27	12.27454	0.00007
Uvula	R	372	6	−70	−29	14.59243	0.00002
Cerebellar tonsil	L	195	−6	−52	−32	9.38866	0.00045
Cerebellar tonsil	R	113	3	−59	−31	8.41985	0.00089

PCu, Precuneus; SPL, Superior Parietal Lobule; FG, Fusiform Gyrus; MTG, MIiddle Temporal Gyrus; ITG, Inferior Temportal Gyrus; SGL, Sub Gyral.

We next investigated whether functional signals in these regions change from learning frequency (Level 0) to learning context-based statistics (Level 1) over time. In particular, we compared fMRI responses for structured and random sequences before and after training for each level (Level 0 vs Level 1) separately. For each participant and brain region identified by the GLM analysis, we calculated normalized fMRI responses [i.e., percentage signal change (PSC) index]; that is, we subtracted mean fMRI responses to random sequences from mean fMRI responses to structured sequences and divided by the average fMRI responses to random sequences. Note that this PSC analysis is complementary to the GLM analysis used to define regions of interest (ROI); it was conducted separately for each level, whereas the GLM tested for differences across sessions (i.e., Pre, Post0, Post1) rather than levels.

Comparing normalized fMRI responses before and after training for Level 0 (Fig. 3*b*) showed that bilateral dorsal frontal regions (medial: SFG: superior frontal gyrus; MeFG: medial frontal gyrus, lateral: MFG: middle frontal gyrus, PrG: precentral gyrus, and IFG: inferior frontal gyrus) and right posterior parietal regions (IPL: inferior parietal lobule, AnG; angular gyrus, and SMG: supramarginal gyrus) were involved in learning frequency-based statistics. These regions showed increased fMRI responses to structured sequences during the pretraining scanning session in contrast to decreased responses after training (i.e., posttraining scanning session). In particular, a repeated-measures ANOVA with session (Pre, Post) and ROI showed a significant main effect of session in the frontal (*F*_(1,20)_ = 7.59, *p* = 0.012) and posterior parietal (*F*_(1,20)_ = 6.58, *p* = 0.018) regions.

In contrast, learning context-based statistics (Level 1) engaged dorsal medial frontal (SFG and MeFG), limbic (CG: cingulate gyrus, ACC: anterior cingulate cortex), and subcortical (Pu: Putamen) areas (Fig. 3*b*). Similar to the fMRI activation patterns for Level 0, dorsal frontal regions showed enhanced responses to structured compared with random sequences for the pretraining scan that decreased after training. This was supported by a repeated-measures ANOVA that showed a significant session effect (frontal: *F*_(1,20)_ = 6.36, *p* = 0.020; limbic: *F*_(1,20)_ = 5.36, *p* = 0.031). In contrast, we observed the opposite pattern of results in putamen (paired *t* test, *t*_(20)_ = −3.31, *p* = 0.003); that is, enhanced activations for structured sequences after training. Activation patterns differed significantly between putamen and frontal limbic regions (i.e., significant interactions of region and session: frontal vs putamen, *F*_(1,20)_ = 16.22, *p* < 0.001; limbic vs putamen, *F*_(1,20)_ = 16.34, *p* < 0.001). In a complementary analysis to the GLM analysis, comparing activations across levels showed significant differences in prefrontal regions (interaction of session and level, *F*_(1,20)_ = 4.83, *p* = 0.040), right posterior parietal regions (main effect of level, *F*_(1,20)_ = 7.41, *p* = 0.013) and putamen (main effect of level, *F*_(1,20)_ = 4.56, *p* = 0.045). Consistent with the GLM analysis, these results support differential involvement of frontoparietal and striatal regions in learning frequency compared with context-based statistics.

Interestingly, the GLM analysis showed activation changes across sessions in the visual cortex (IOG: inferior occipital gyrus, MOG: middle occipital gyrus, LiG: lingual gyrus). Comparing fMRI responses in these regions across sessions did not show any significant differences for either of the two levels (Level 0: *F*_(1,20)_ < 1, *p* = 0.429; Level 1: *F*_(1,20)_ < 1, *p* = 0.531), suggesting that fMRI responses for structured sequences did not change significantly with training in the visual cortex. For learning frequency statistics (Level 0) visual cortex showed stronger activations for random than structured sequences (i.e., negative PSC index values) both before (main effect of sequence, *F*_(1,20)_ = 6.04, *p* = 0.023) and after (*F*_(1,20)_ = 32.7, *p* < 0.001) training, suggesting decreased activation due to repetition (i.e., repetition suppression) of symbols that appeared more frequently in structured than random sequences (Summerfield and Egner, 2009). This effect was not observed for first-order sequences (Level 1; before training, *F*_(1,20)_ < 1, *p* = 0.981; after training, *F*_(1,20)_ = 1.87, *p* = 0.187), consistent with higher repetition of single symbols in zero-order than first-order sequences.

**Figure 4.**
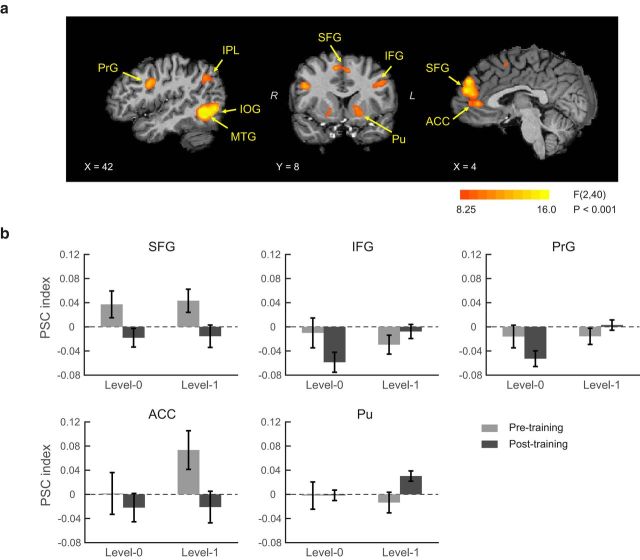
fMRI results controlled for differences in sequence entropy across levels. ***a***, GLM maps (*p* < 0.001, cluster threshold corrected) for 2-way interaction between scanning session (Pre, Post0, Post1) and sequence (structured vs random) including entropy rate as a regressor. ***b***, PSC index before and after training for Level 0 and Level 1. Error bars indicate SEM. Data are shown for ROIs that showed a significant interaction between session (pre vs posttraining) and sequence (structured vs random).

Next, we asked whether the differences we observed in the activation patterns between levels were due to differences in sequence predictability. To measure sequence predictability, we computed the entropy rate of the probability distribution of all possible sequences. For Level 0, the entropy rate is defined as the entropy of the stationary distribution of symbols in the sequence. For Level 1, the entropy rate is a weighted sum of the entropies of all context-conditional distributions where the weights are given by the stationary distribution of contexts. We calculated the entropy rate for each sequence; we then conducted the whole brain voxelwise GLM analysis using entropy rate as regressor. This analysis showed significant interactions (*p* < 0.001, cluster threshold corrected) between sequence (structured vs random) and scanning session (Pre, Post0, Post1) in similar regions as the main analysis (Fig. 4*a*), making it unlikely that our results were confounded by differences in sequence predictability between levels.

Comparing normalized fMRI responses before and after training (Fig. 4*b*) for Level 0, we observed increased fMRI responses to structured sequences before than after training (*F*_(1,20)_ = 5.18, *p* = 0.034) in bilateral frontal regions (SFG; PrG, and IFG). In contrast, learning context-based statistics (Level 1) engaged dorsal frontal (SFG), limbic (ACC) and subcortical (putamen) areas. Dorsal frontal and limbic regions showed enhanced responses to structured compared with random sequences for the pretraining scan that decreased after training (*F*_(1,20)_ = 5.76, *p* = 0.026). In contrast, putamen showed enhanced activations for structured sequences after training (paired *t* test, *t*_(20)_ = −2.78, *p* = 0.012). Activation patterns differed significantly between putamen and frontal limbic regions (i.e., significant interactions of region and session: *F*_(1,20)_ = 13.9, *p* < 0.001), in support of differential involvement of frontal and striatal regions in learning temporal statistics.

Our results so far suggest that dorsal corticostriatal mechanisms mediate learning of behaviorally relevant statistics. In particular, frontoparietal and cingulate regions showed higher fMRI responses for structured than random sequences during the pretraining scan. This is consistent with the role of dorsal prefrontal cortex in decision making (Heekeren et al., 2008; Rushworth and Behrens, 2008) and predictive coding (Monchi et al., 2001; Bar, 2009); that is, processes that are involved in both learning of frequency and context-based statistics. Further, our results show that cingulate cortex is involved in learning more complex context-based statistics that may relate to its involvement in learning under increased uncertainty (Kahnt et al., 2011; Nastase et al., 2014). Higher fMRI responses for structured sequences in these regions at the beginning of training may reflect processing of novel structures (i.e., temporal regularities in the form of single- or paired-item repetition). Significantly higher performance for structured sequences than random guessing during the first scanning session suggests that participants extract these statistics early in the training. Interestingly, fMRI responses for structured sequences decreased as these sequences became familiar with training. This decreased signals can be understood in the context of repetition suppression previously observed for predictable events (Raichle et al., 1994; den Ouden et al., 2009; Summerfield and Egner, 2009; Alink et al., 2010; Kok et al., 2012). In contrast, dorsal striatal regions (i.e., putamen), which have been implicated in learning probabilistic associations (Rauch et al., 1997; Poldrack and Packard, 2003), showed higher fMRI responses for structured compared with random sequences after training with first-order sequences, suggesting that representations of context–target contingencies were acquired through training.

### Control analyses

We conducted a number of additional analyses and experiments to help rule out alternative explanations of our results.

First, we investigated whether the differences that we observed in fMRI responses between structured and random sequences were due to the participants attending more to the structured sequences either as the novel stimulus before training or the familiar stimulus after training. Comparing response times to structured and random sequences in the pretraining and posttraining session (3-way mixed-design ANOVA: session × sequence × level) showed decreased response times after training (main effect of session: *F*_(1,20)_ = 8.63, *p* = 0.008), but no significant differences between structured and random sequences (main effect of sequence, *F*_(1,20)_ = 0.152, *p* = 0.700), suggesting that participants engaged with the task when both structured and random sequences were presented. Importantly, there was no significant interaction among session, sequence, and level (*F*_(1,20)_ = 1.72, *p* = 0.205), suggesting that differences in activation patterns across levels could not be simply due to differences in attention or task difficulty. Further, analysis of eye movement data collected during scanning did not show any significant differences between structured and random sequences for Level 0 or Level 1. There were no significant interactions observed (*p* > 0.10), suggesting that it is unlikely that our findings were confounded significantly by eye movements.

Second, we tested whether the learning-dependent fMRI changes that we observed could be confounded by differences in the number of training sessions across participants. Training duration varied from three to five sessions per level across participants, with most participants completing four training sessions (Level 0, *n* = 12; Level 1, *n* = 17) before reaching plateau performance. An ANCOVA analysis on the behavioral data using the number of training sessions as covariate did not show any significant interactions between session and number of training sessions (Level 0: *F*_(1,19)_ = 0.479, *p* = 0.497; Level 1: *F*_(1,19)_ = 0.089, *p* = 0.768). Similar analysis on the fMRI data did not show any significant interaction between session and number of training sessions (Level 0: frontal, *F*_(1,19)_ = 0.001, *p* = 0.874, parietal, *F*_(1,19)_ = 0.447, *p* = 0.512; Level 1: frontal, *F*_(1,19)_ = 0.473, *p* = 0.500, limbic, *F*_(1,19)_ = 0.705, *p* = 0.412, subcortical regions, *F*_(1,19)_ = 3.53, *p* = 0.076). Together, these analyses suggest that it is unlikely that our fMRI results were confounded by differences in training duration across participants.

**Figure 5.**
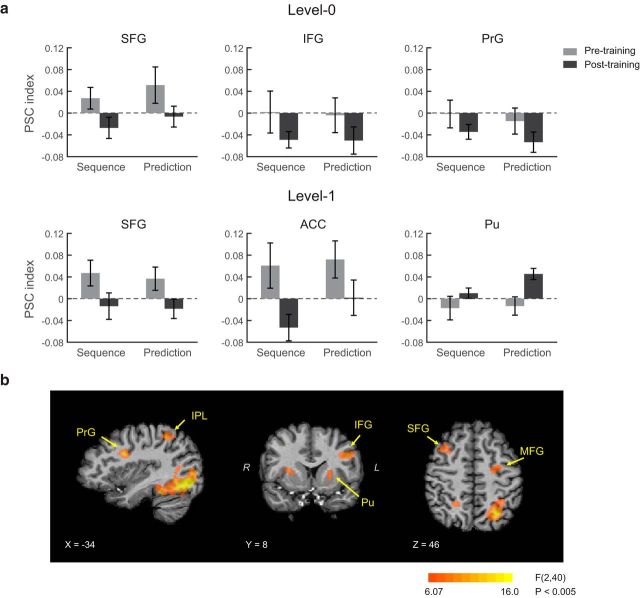
fMRI results for sequence presentation and participants' prediction. ***a***, PSC index for sequence presentation (volumes 1–2) and participant prediction (volumes 4–5) before and after training for Level 0 and Level 1. Data are shown for the representative ROIs from Figure 4*b*. Error bars indicate SEM. ***b***, GLM maps for the 2-way interaction between scanning session and sequence at *p* < 0.005 (cluster threshold corrected) using only the volumes that correspond to sequence presentation.

Third, we investigated whether the activation patterns that we observed relate to learning-dependent changes in the representation of the trained sequences or simply the participants' responses. In our design, the ISI jitter in each trial is too short to isolate the fMRI signal per stimulus in the sequence. However, the design of the paradigm allows us to analyze our fMRI data related to sequence presentation separately from participant prediction. First, we compared PSC for the first two volumes related to the presented sequences and the fourth and fifth volume related to the participants' prediction (i.e., the third volume was not included in this analysis because the sequences lasted 2.5 volumes). This analysis (Fig. 5*a*) showed that activation patterns for fMRI signals related to the sequence presentation and the participants' prediction were similar to those observed in our main analysis (Figs. 3*b*, 4*b*). In particular, we observed a significant effect of session (i.e., pre vs posttraining; Level 0: frontal: *F*_(1,20)_ = 4.97, *p* = 0.037; Level 1: frontal limbic: *F*_(1,20)_ = 5.95, *p* = 0.024, putamen: *F*_(1,20)_ = 7.29, *p* = 0.014), but no significant effect of processing stage (i.e., sequence vs prediction; Level 0: frontal: *F*_(1,20)_ = 0.004, *p* = 0.951: Level 1: frontal limbic: *F*_(1,20)_ = 0.399, *p* = 0.535: putamen: *F*_(1,20)_ = 3.29, *p* = 0.085). There was no significant interaction of session and processing stage (Level 0: frontal: *F*_(1,20)_ = 0.003, *p* = 0.954; Level 1: frontal limbic: *F*_(1,20)_ = 0.496, *p* = 0.490; putamen: *F*_(1,20)_ = 1.68, *p* = 0.209). Second, a whole-brain voxelwise GLM analysis using only the volumes that corresponded to the sequence presentation showed significant interactions (*p* < 0.001, cluster threshold corrected) between sequence (structured vs random) and scanning session (Pre, Post0, Post1) in similar regions as the main analysis (Fig. 5*b*). Together, these analyses of fMRI signals related to the sequence presentation showed similar activation patterns as the main analysis (Fig. 3*a*), which included fMRI signals from both the sequence presentation and the participant prediction. Therefore, the learning-dependent changes that we observed in the main analysis relate to the sequence structure and could not be simply driven by the participants' prediction or response because fMRI signals related to the sequence presentation were recorded before the participants responded to the test stimulus.

### Response strategies: matching versus maximization

Previous work (Shanks et al., 2002; Rieskamp and Otto, 2006; Eckstein et al., 2013; Acerbi et al., 2014; Fulvio et al., 2014; Murray et al., 2015) on probabilistic learning and decision making has proposed that individuals use two possible response strategies when making a choice: matching and maximization. Observers have been shown to either match their choices stochastically according to the underlying input statistics or to maximize their reward by selecting the most probable positively rewarded outcomes. In the context of our task, because the Markov models that generated stimulus sequences were stochastic, participants needed to learn the probabilities of different outcomes to succeed in the prediction task. It is possible that participants used probability maximization in which they always select the most probable outcome in a particular context. Alternatively, participants might learn the relative probabilities of each symbol [e.g., p(A) = 0.18, p(B) = 0.72, p(C) = 0.05, p(D) = 0.05)] and respond so as to reproduce this distribution, a strategy referred to as probability matching.

**Figure 6.**
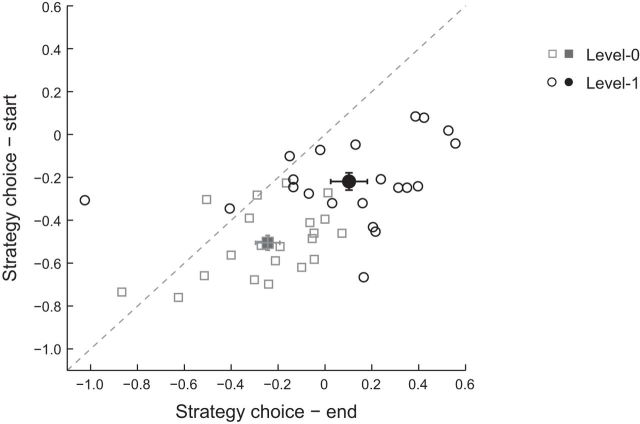
Strategy choice. Strategy choice is shown at the beginning (first two runs) and end (last two runs) of training for Level 0 (squares) and Level 1 (circles). Open symbols indicate individual participant data; closed symbols indicate mean date per level. Strategy choice was measured by comparing participant responses to two possible strategies: matching (i.e., predicting the presented target distribution) versus maximization (i.e., predicting the high probability targets per context). Negative values indicate a strategy closer to matching, whereas positive values indicate a strategy closer to maximization. Error bars indicate SEM.

To quantify the participants' strategies, we computed a strategy index that indicates participant's preference (on a continuous scale) for responding using probability matching versus maximization. Figure 6 illustrates individual strategy at the beginning (first two blocks) and end (last two blocks) of training. Comparing individual strategy across levels showed significantly higher values after training for Level 1 compared with Level 0 (*F*_(1,20)_ = 26.2, *p* < 0.001). This shift in individual strategy was evident mainly after training (*F*_(1,20)_ = 35.8, *p* < 0.001); that is, participants shifted more toward maximization when learning context-based rather than frequency statistics. Note that this relationship was not confounded by differences in performance because there were no significant correlations (Level 0: *r* = 0.31, *p* = 0.17; Level 1: *r* = 0.22, *p* = 0.34) of PI at the end of training (mean PI for the last two blocks of training) and strategy index. Interestingly, despite greater maximization for more complex structures than frequency statistics, we note that participants did not achieve optimal maximization performance. Maximization is typically observed under supervised or reinforcement learning paradigms (Shanks et al., 2002), so it is perhaps not surprising that our participants did not achieve exact maximization because trial-by-trial feedback was not provided.

### fMRI covariance analysis with strategy

To investigate the relationship between brain activations and individual strategy, we conducted a voxelwise GLM covariance analyses. In particular, we correlated learning-dependent changes in fMRI signal (Posttraining PSC minus Pretraining PSC) for structured (compared with random) sequences with individual strategy. We calculated a Pearson correlation coefficient (*R*) for each voxel across the whole brain and identified voxel clusters showing significant correlations (*p* < 0.05) for learning frequency (Level 0) and context-based statistics (Level 1), respectively. Positive correlations indicate increased activations after training that relate to maximization, whereas negative correlations indicate increased activations after training that relate to matching because negative strategy values indicate strategy toward matching.

**Figure 7.**
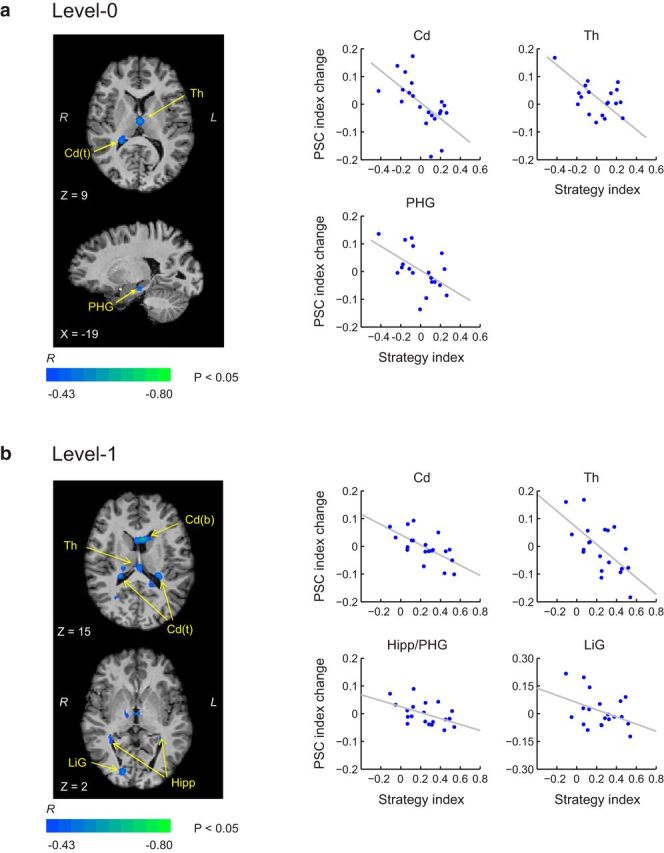
Brain activations correlating with matching. Covariance analysis shows significant (*p* < 0.05, cluster threshold corrected) negative correlations (*R* correlation coefficient) between individual strategy index and learning-dependent fMRI change (i.e., after vs before training) for Level 0 (***a***) and Level 1 (***b***). Whole-brain maps and plots show negative correlations between strategy index and PSC index change (posttraining vs pretraining) for representative ROIs, as derived from the covariance analysis (note that these correlation plots are only presented for demonstration purposes; no additional statistical analysis was performed in these ROIs after the covariance analysis to avoid circularity). Cd, Caudate: b, body; t, tail; Th, thalamus; PHG, parahippocampal gyrus; Hipp, hippocampus.

First, we observed negative correlations between learning-dependent fMRI changes and strategy index in occipitotemporal (including hippocampal regions), basal ganglia (ventral caudate), and thalamic regions (Fig. 7). These correlations indicate that increased activations for structured sequences after training in these regions relate to matching. Further, these correlations were observed for both levels, suggesting that learning frequency or context-based statistics by matching involves regions in visual corticostriatal circuits that have been implicated previously in the implicit learning of temporal sequences (Hindy et al., 2016; Rosenthal et al., 2016) and novel categories (Ashby and Maddox, 2005; Seger, 2013). In particular, previous work has implicated the striatum and the medial temporal lobe (i.e., hippocampus; Rauch et al., 1997; Poldrack and Packard, 2003; Schendan et al., 2003; Cools et al., 2004; Gheysen et al., 2011; Rose et al., 2011; Schapiro et al., 2012; Hsieh et al., 2014) in learning probabilistic associations. Further, medial temporal cortex has been implicated in explicit rule-based categorization, whereas caudate in categorization based on information integration (Nomura et al., 2007).

**Figure 8.**
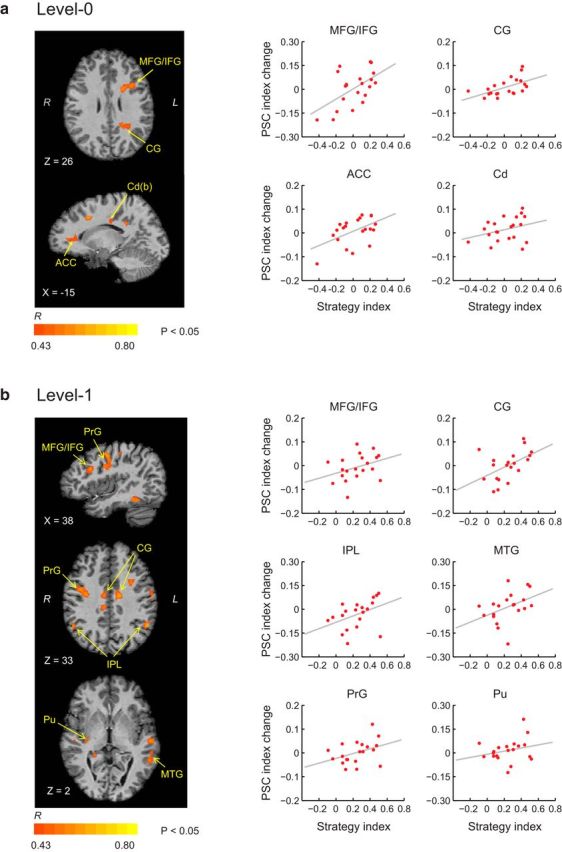
Brain activations correlating with maximization. Covariance analysis shows significant (*p* < 0.05, cluster threshold corrected) positive correlations (*R* correlation coefficient) between individual strategy index and learning-dependent fMRI change (i.e., posttraining vs pretraining) for Level 0 (***a***) and Level 1 (***b***). Whole-brain maps and plots show positive correlations between strategy index and PSC index change (posttraining vs pretraining) for representative ROIs, as derived from the covariance analysis (note that these correlation plots are only presented for demonstration purposes; no additional statistical analysis was performed in these ROIs after the covariance analysis to avoid circularity).

In contrast, we observed positive correlations between learning-dependent fMRI changes and strategy index, indicating that increased activations for structured sequences after training relate to maximizing (Fig. 8). In particular, for Level 0, we observed positive correlations in dorsolateral prefrontal areas (MFG/IFG), the dorsal caudate and the cingulate (including anterior cingulate) cortex. For Level 1, we observed positive correlations in dorsolateral prefrontal (MFG/IFG) and posterior parietal regions, as well as cingulate and temporal cortex. Interestingly, we also observed positive correlations for sensory–motor cortex (precentral and postcentral gyrus) and basal ganglia (putamen). Our results are consistent with the role of prefrontal and cingulate cortex in decision making, monitoring performance, correcting errors, and switching between associations and strategies. Previous work on humans and animals emphasizes the role of the caudate in switching between strategies (Monchi et al., 2001; Cools et al., 2004; Seger and Cincotta, 2006) and learning after a rule reversal (Cools et al., 2002; Pasupathy and Miller, 2005). This tonic and fast learning in the caudate is thought to train slower learning mechanisms in the frontal cortex that may facilitate generalization and abstraction of learned associations. Finally, the putamen, which is known to be involved in skilled and habitual performance (Daw et al., 2005; Balleine and O'Doherty, 2010), may facilitate learning by maximizing. That is, once participants have extracted the most probable outcome for a given context, they may then select it habitually as the predicted outcome.

## Discussion

Here, we investigated the brain mechanisms that medicate our ability to adapt to changes in the environment's statistics and make predictions. To test how individuals extract structure changes, we manipulated the complexity of temporal sequences during training from simple frequency to context-based statistics. Our results provide evidence for dissociated corticostriatal mechanisms that mediate our ability to extract behaviorally relevant statistics. We found that frontoparietal activity decreases for frequency-based learning, whereas context-based learning is associated with decreased frontocingulate activity and increased striatal activity. Decreased fMRI signals in frontoparietal circuits can be understood in the context of predictive coding as repetition suppression for predictable events (Raichle et al., 1994; den Ouden et al., 2009; Summerfield and Egner, 2009; Alink et al., 2010; Kok et al., 2012). In contrast, increased fMRI signals in putamen, which are implicated in learning probabilistic associations (Rauch et al., 1997; Poldrack and Packard, 2003), suggest representations of predictive structures acquired through training.

Importantly, our approach allows us to track participants' predictions and their decision strategies during training. We demonstrated that learning predictive structures relates to decision strategies; that is, learning complex structures relates to extracting the most probable target per context (i.e., maximizing) than matching the exact sequence statistics. Importantly, these decision strategies engage distinct corticostriatal circuits: performance based on probability matching engages occipitotemporal and basal ganglia (ventral caudate) regions, whereas performance based on maximizing engages dorsolateral prefrontal, cingulate, sensory–motor regions, and basal ganglia (dorsal caudate, putamen). Recent work has focused on the role of the hippocampus in learning temporal sequences (Hsieh et al., 2014; Rosenthal et al., 2016) and predictive associations (Hindy et al., 2016). Our findings suggest an alternate route to learning via maximizing that is implemented by interactions between executive and motor corticostriatal mechanisms rather than visual corticostriatal circuits (including hippocampal cortex) that support learning by matching.

Previous studies have implicated these corticostriatal circuits in reinforcement learning (for reviews, see Robbins, 2007; Balleine and O'Doherty, 2010). We show here that learning predictive statistics may proceed without explicit trial-by-trial feedback and involve interactions between corticostriatal circuits similar to those known to support reward-based learning (Alexander et al., 1986; Lawrence et al., 1998). In particular, we show that dorsal frontoparietal regions are involved in extracting novel regularities, monitoring and adjusting strategy throughout training. In contrast, striatal regions represent context-based statistics learned through bootstrap training (i.e., multiple sessions of exposure to structured sequences) that may optimize the selection of the most probable outcome in a given context. Previous work investigating learning of sequential contingencies in the context of the serial reaction time task suggests that striatal versus hippocampal circuits relate to distinct error-driven learning processes and operate at different learning rates (Bornstein and Daw, 2012). In particular, fast learning was shown to engage striatal regions (i.e., putamen), whereas slow learning engages the hippocampus. Although our paradigm does not dissociate learning time course from structure complexity, it is possible that learning of temporal structures proceeds from corticostriatal to hippocampal circuits.

Further, we considered whether the learning that we observed occurred in an incidental manner or involved explicit knowledge of the underlying sequence structure. Previous studies have suggested that learning of regularities may occur implicitly in a range of tasks: visuomotor sequence learning (Nissen and Bullemer, 1987; Seger, 1994; Schwarb and Schumacher, 2012), artificial grammar learning (Reber, 1967), probabilistic category learning (Knowlton et al., 1994), and contextual cue learning (Chun and Jiang, 1998). This work has focused on implicit measures of sequence learning, such as familiarity judgments or reaction times. In contrast, our paradigm allows us to test directly whether exposure to temporal sequences facilitates the observers' ability to predict the identity of the next stimulus in a sequence explicitly. Although, our experimental design makes it unlikely that the participants memorized specific stimulus positions or the full sequences, debriefing the participants suggests that most extracted some high-probability symbols or context–target combinations. Therefore, it is possible that prolonged exposure to probabilistic structures (i.e., multiple sessions in contrast to single exposure sessions typically used in statistical learning studies) in combination with prediction judgments (Dale et al., 2012) may evoke some explicit knowledge of temporal structures, in contrast to implicit measures of anticipation typically used in statistical learning studies.

Finally, previous work has implicated additional brain regions related to learning modality-specific regularities (Nastase et al., 2014); that is, visual cortex is implicated in learning visual statistical regularities (Aizenstein et al., 2004; Turk-Browne et al., 2010; Meyer and Olson, 2011), whereas inferior frontal and temporal regions in learning temporal regularities related to music and language (Bahlmann et al., 2009; Leaver et al., 2009; Karuza et al., 2013; Koelsch et al., 2013). Our results provide evidence for corticostriatal mechanisms that mediate learning of predictive statistics. We speculate that these mechanisms may mediate domain-general learning of complex structures that can be specialized to support higher cognitive functions such as learning music or language.
